# Editorial: Roles of gut microbiota in cancers of the gastrointestinal tract

**DOI:** 10.3389/fmicb.2025.1585090

**Published:** 2025-04-07

**Authors:** Sunny H. Wong, William K. K. Wu

**Affiliations:** ^1^Lee Kong Chian School of Medicine, Nanyang Technological University, Singapore, Singapore; ^2^Department of Gastroenterology and Hepatology, Tan Tock Seng Hospital, National Healthcare Group, Singapore, Singapore; ^3^Department of Anaesthesia and Intensive Care, Peter Hung Pain Research Institute, The Chinese University of Hong Kong, Hong Kong, Hong Kong SAR, China; ^4^State Key Laboratory of Digestive Disease, Li Ka Shing Institute of Health Sciences, The Chinese University of Hong Kong, Hong Kong, Hong Kong SAR, China

**Keywords:** microbiome, gastric cancer, liver cancer, esophageal cancer, pancreatic cancer

The gut microbiota is a dynamic ecosystem residing within the human gastrointestinal tract, and is progressively recognized as a crucial factor influencing cancer initiation, progression, and treatment response. Mounting evidence underscores the microbiota's role as a key regulator of host immunity, metabolism, and inflammation, which are pathways linked to carcinogenesis. Of particular interest is the microbiota's involvement in digestive cancers, including colorectal cancer (CRC), gastric cancer, and esophageal cancer, where microbial dysbiosis has been consistently reported (Garrett, 2019; Wong and Yu, 2019). Despite extensive research into CRC microbiota, the gastric and esophageal microbiotas were relatively less studied until recently; however, these areas are now garnering increased research attention as potential sources of novel biomarkers and therapeutic targets.

## Research trend and microbial pathogenesis in digestive cancers

A recent bibliometric analysis by Ke et al. identifies a clear and accelerating trend toward investigating the gastric microbiota in gastric cancer research. This comprehensive study reveals a notable shift from the previously dominant focus on *Helicobacter pylori* alone toward broader exploration of the gastric microbiota, including emerging non-*Helicobacter* bacteria (e.g., *Fusobacterium nucleatum, Streptococcus anginosus*). In this connection, *F. nucleatum* could promote immune evasion in gastric cancer via recruiting tumor-associated neutrophils while *S. anginosus* could promote gastric tumorigenesis through the Annexin A2-mitogen-activated protein kinase axis (Zhang et al., 2025; Fu et al., 2024). The study by Ke et al. highlights the increasing emphasis on elucidating the microbial mechanisms underlying gastric carcinogenesis. Similarly, a systematic review of case-control studies by Zhang R. et al. provides evidence that gastric cancer patients harbor distinct microbial signatures, such as increased *Lactobacillus spp*. and *Streptococcus spp*. and decreased *Porphyromonas spp*. and *Rothia spp*.. Such findings underscore the necessity for consensus microbial signatures across diverse populations and methodologies, reflecting the complexity and specificity of microbiota alterations in cancer.

To move beyond observational associations toward establishing causality, increased efforts are focusing on methodologies such as Mendelian randomization. Recent analyses by Ma et al. for CRC and Zhang Z. et al. for esophageal cancer have pinpointed potentially causal microbial taxa in carcinogenesis. Ma et al. demonstrated the positive associations of *Porphyromonadaceae spp., Lachnospiraceae UCG010, Lachnospira*, and *Sellimonas* with CRC. Notably, although *Lachnospiraceae UCG010* exhibited a negative correlation with interleukin-10, the analysis suggested that the CRC-promoting effect of *Lachnospiraceae UCG010* was independent of this cytokine. Likewise, Zhang Z. et al. identified the negative associations of *Romboutsia, Lachnospira*, and *Eubacterium* with esophageal cancer. These protective microbes might protect against esophageal cancer formation through enhancing cellular resistance to endoplasmic reticulum stress, inhibiting inflammatory responses, and scavenging free radicals. The authors also identified the potential pathogenic role for *Veillonella* in esophageal cancer. To this end, this bacterium has been shown to promote inflammatory responses via activating the Toll-like receptor 4 pathway in macrophages. These studies reinforce the importance of distinguishing correlation from causation, guiding the identification of microbial culprits, and refining targets for microbiota-based interventions.

While colorectal microbiota research remains robust, the previously less-explored gastric and esophageal microbiotas are now emerging as some areas of research focus. Future efforts must aim for cross-population validation, interdisciplinary collaboration and biological insight to fully harness the microbiota's potential for translation.

## Microbiota-based biomarkers for digestive cancers

Over the last decade, the scientific community has witnessed a rapid expansion in the number of studies utilizing gut microbes as biomarkers for the detection of neoplastic lesions, especially those of the digestive organs. In this Research Topic, Cui et al. introduces a deep learning model named multi-view convolutional variational information bottleneck (MV-CVIB) for predicting metastatic colorectal cancer (mCRC) using 16S rDNA sequencing-based gut microbiota data. The model integrates microbial abundance data with nearest neighbor information, achieving an area under the receiver operating characteristic curve (AUROC) of >0.9 on the mCRC dataset and demonstrating good performance for distinguishing CRC patients from healthy subjects on two additional CRC datasets (AUROC = 0.82 and 0.83, respectively). The study also identified significant microbial differences between mCRC and non-mCRC patients, particularly the enrichment of *Propionibacterium acnes* in the former. MV-CVIB thus represents a new deep learning tool for microbiota-based disease classification. Similarly, Zhou et al. compared the microbial communities in mCRC and non-mCRC, but they focused on tissue-associated instead of luminal bacteria. The researchers found that mCRC was characterized by an increase in *Bacteroides*, particularly *B. fragilis* and *B. uniformis*, and a decrease in *Streptococcus*. Interestingly, microbial differences in tumor-adjacent tissues from mCRC and non-mCRC persisted, indicating that a microbial “field defect” might contribute to CRC metastasis. In terms of the classification performance, these bacteria only exhibited a modest accuracy (AUROC = 0.64 for *Bacteroides* or *Streptococcus*) but their combination with carcinoembryonic antigen (CEA) improved the prediction (AUROC = 0.71 for CEA + *Streptococcus*). This study again suggests a potential role of the gut microbiota in CRC metastasis. Unlike the role in CRC, authors of another article in this Research Topic identified the enrichment of *Streptococcus* in the gut microbiota among patients with pancreatic cancer (PC), especially those with liver metastasis (PCLM) (Yang et al.). In this respect, *Streptococcus* could discriminate PC patients and PCLM patients from healthy subjects and non-metastatic PC patients, respectively, with high accuracy (AUROC = 0.93 for PC; AUROC = 0.80 for PCLM).

Different from the bacteriome, the use of plasmids (small circular, non-chromosomal DNA molecules found in bacteria) as biomarkers has been understudied. In this connection, Cai et al. examined the potential of using gut plasmids as novel diagnostic biomarkers for CRC. By analyzing metagenomic data from over 1,200 samples across eight cohorts, the researchers identified 198 plasmid sequences differentially abundant in CRC patients. A diagnostic model using 21 plasmid markers achieved a moderate accuracy with an AUROC of 0.70. Combining the plasmid markers with the bacterial markers further improved the accuracy (mean AUROC = 0.80). These findings underscore the utility of plasmids in enhancing diagnostic models despite the current challenges in their detection from short-read sequencing data.

## Microbiota-based therapeutics for digestive cancers

Inflammatory bowel disease, including ulcerative colitis, is associated with an increased risk of CRC whereas the traditional Chinese medicine maggot has demonstrated anti-inflammatory properties in other disease contexts. In the work by Tang et al., maggot extract was shown to reverse the alterations of the gut microbiota and the associated metabolome in a murine model of colitis-associated CRC. Importantly, the reversal of dysbiosis was accompanied by the improvement of gut barrier function and the alleviation of inflammatory signals, hinting at the therapeutic potential of the restoration of a healthy gut microbiota in preventing colitis-associated CRC. Feng et al. also elegantly summarize how the gut microbiota might interact with the tumor microenvironment that has a direct impact on the therapeutic response, especially in the context of cancer immunotherapy. For instance, *Bifidobacterium pseudolongum* could drive T helper 1 cell differentiation and its high abundance is associated with response to immune checkpoint inhibitors. Short-chain fatty acids produced by commensals can also promote the memory potential of activated CD8^+^ T cells, contributing to the responsiveness to immune checkpoint inhibitors.

## Concluding remarks

This Research Topic represents a notable collection of articles highlighting the current research trends in the field of gut microbiota in cancers of the gastrointestinal tract. Pathogenic roles of specific gut microbes have been scrutinized by systematic reviews and Mendelian randomization. With clinical relevance, several articles highlight the potential of using gut microbial biomarkers for non-invasive screening of gastrointestinal cancers and predicting their metastasis. However, the small sample size of some of these studies may raise concerns about overfitting. Their retrospective design also limits the generalizability. Larger-cohort validation, particularly across geographic regions and with subjects recruited in a prospective manner, is thus needed to confirm the clinical utility. Therapeutically, manipulating the gut microbiota might hold promise for preventing gastrointestinal cancers and improving response to systemic therapies.
